# Prediction of early myocardial damage in obstructive sleep apnea patients using combined logistic regression and QUEST decision tree models

**DOI:** 10.1590/1414-431X2025e14757

**Published:** 2025-06-20

**Authors:** Chong Pei, Zhen Ding, Lei Hu, Shuyu Gui

**Affiliations:** 1 Department of Respiratory and Critical Care Medicine, The First Affiliated Hospital of Anhui Medical University, Hefei, Anhui, China; 2 Department of Respiratory and Critical Care Medicine, The Third Affiliated Hospital of Anhui Medical University (The First People's Hospital of Hefei), Hefei, Anhui, China; 3 The First Affiliated Hospital of USTC, Division of Life Sciences and Medicine, University of Science and Technology of China, Hefei, Anhui, China

**Keywords:** Obstructive sleep apnea, LVGLS, RVFWLS, Logistic regression, QUEST decision tree

## Abstract

Obstructive sleep apnea (OSA) is linked to cardiovascular complications, including myocardial dysfunction, yet early detection remains difficult. This retrospective study aimed to develop a combined logistic regression and QUEST decision tree model to predict early myocardial dysfunction in OSA patients. Echocardiography left ventricular global longitudinal strain (LVGLS) and right ventricular free wall longitudinal strain (RVFWLS) were used to assess myocardial function in OSA patients. Predictive models were constructed using clinical parameters. External validation involved 100 OSA patients from a respiratory sleep clinic. LVGLS and RVFWLS were significantly impaired in OSA patients, particularly in moderate-to-severe cases. BMI, percentage of sleep time with oxygen saturation <90% (CT90%), and arterial bicarbonate were identified as key predictors. The combined model achieved superior predictive accuracy, with an area under the curve of 0.91 for LVGLS and RVFWLS reductions, outperforming individual models. External validation confirmed the stability and generalizability of the model. The combined logistic regression and QUEST decision tree model accurately predicted early myocardial dysfunction in OSA patients, providing a valuable tool for personalized risk assessment and early intervention.

## Introduction

Obstructive sleep apnea (OSA) is a common sleep-related breathing disorder characterized by recurrent episodes of apnea and hypopnea during sleep, leading to intermittent hypoxia, oxidative stress, and inflammatory responses (1). These pathological mechanisms are known to exert significant effects on the cardiovascular system, particularly contributing to myocardial injury. Studies have shown that patients with OSA have an increased risk of developing cardiovascular diseases, such as hypertension, heart failure, and coronary artery disease, with myocardial injury often being an early manifestation of these complications (2,3). Early identification and prediction of myocardial damage in OSA patients are critical for reducing the incidence of cardiovascular events and improving patient prognosis.

Early myocardial damage in patients with OSA is often difficult to detect through routine clinical examinations. Studies have shown that myocardial longitudinal strain parameters are sensitive indicators for the early identification of myocardial dysfunction (4,5). The current gold standard for diagnosing OSA is overnight polysomnography (PSG), which classifies disease severity based on the apnea-hypopnea index (AHI) (1). However, while PSG can evaluate OSA severity, it is limited in its ability to predict early myocardial dysfunction. As a result, some patients with mild or moderate OSA may have undetected myocardial damage, potentially delaying timely clinical interventions. Early prediction of myocardial injury not only facilitates better disease assessment but also helps guide treatment strategies, such as the use of continuous positive airway pressure (CPAP), thereby more effectively preventing the progression of cardiovascular complications and potentially even reversing early myocardial functional impairment (6,7).

To address this gap, this study proposes a combined predictive model for early myocardial injury in OSA patients, integrating multivariate logistic regression (LR) and the Quick, Unbiased, Efficient Statistical Tree (QUEST) decision tree model. Logistic regression quantifies the risk factors for myocardial injury and their correlations, while the QUEST model captures nonlinear features and complex interactions. QUEST is a decision tree algorithm that uses statistical tests to select the best predictive variables and ensure unbiased splits. By utilizing a robust splitting criterion, QUEST reduces overfitting and improves predictive performance, particularly when dealing with datasets with complex variable interactions. By incorporating clinical baseline data, blood test indices, PSG data, and echocardiographic findings, the model aims to identify early signs of myocardial dysfunction in OSA patients.

The objective of this study was to establish and validate a predictive model that may guide clinical decision-making, enabling more personalized CPAP therapy for high-risk OSA patients. This approach is expected to reduce the risk of myocardial damage progression to more severe cardiovascular diseases, ultimately improving long-term health outcomes for OSA patients.

## Material and Methods

### Patients

The study consecutively selected 678 patients who visited the Sleep Center of the Third Affiliated Hospital of Anhui Medical University (Hefei First People's Hospital) and underwent PSG examination between March 2019 and March 2022 as research subjects.

Inclusion criteria were: 1) age over 18 years; 2) normal cognitive function and intact autonomous behavior ability; 3) complete clinical data, including general information, blood biochemical indices, and cardiac ultrasound parameters; 4) complete PSG examination during hospitalization; and 5) ability to independently and accurately complete the questionnaire.

Exclusion criteria were: 1) presence of conditions other than OSA that may affect blood gas analysis results, such as laryngeal diseases, vocal cord disorders, tracheal foreign bodies, chronic obstructive pulmonary disease (COPD), acute exacerbation of bronchial asthma, interstitial lung disease, anemia, electrolyte imbalance, and cardiovascular diseases other than hypertension; 2) presence of chronic diseases that may affect blood HCO_3_^-^ concentration, such as heart and lung failure or liver and kidney dysfunction; 3) use of a ventilator within the past month; 4) presence of other sleep-related breathing disorders aside from OSA; 5) individuals in special conditions or statuses (e.g., pregnancy, lactation, postpartum, or mental disorders); 6) use of sedatives or antipsychotic medications within the past month; 7) use of diuretics; 8) incomplete clinical data; 9) potential abnormal electroencephalogram (EEG) findings (e.g., epilepsy, brain tumors, or deep brain stimulator implants); 10) central respiratory events accounting for more than 50% of total respiratory events; and 11) total nighttime sleep duration of less than 5 h.

This study was approved by the Ethics Committee of the Third Affiliated Hospital of Anhui Medical University (First People's Hospital of Hefei) and was granted a waiver of informed consent.

### General clinical data

Basic demographic and clinical data, including gender, age, height, weight, body mass index (BMI), neck circumference (NC), abdominal circumference (AC), systolic and diastolic blood pressures (SBP, DBP), smoking history, and alcohol consumption, were collected.

### Blood indicators

Fasting blood samples were collected to assess alanine aminotransferase (ALT), triglycerides (TG), total cholesterol (TC), high- and low-density lipoprotein cholesterol (HDL-C, LDL-C), fasting glucose (GLU), bicarbonate (HCO_3_^-^), C-reactive protein (CRP), interleukin-6 (IL-6), and angiotensin I (Ang I).

### PSG recording and analysis

Participants underwent PSG using an Alice 6 device (Philips Respironics, USA). Key parameters including AHI, lowest oxygen saturation (LSaO_2_), and mean oxygen saturation (MSaO_2_) were recorded. Diagnosis followed 2012 Asian Society of Sleep Medicine (ASSM) guidelines for OSA severity classification: AHI 5-15 (mild), 15-30 (moderate), and >30 (severe).

### Arterial blood gas analysis

Arterial blood was collected for blood gas analysis, measuring parameters such as partial pressure of arterial oxygen (PaO_2_), partial pressure of arterial carbon dioxide (PaCO_2_), potential of hydrogen (pH), HCO_3_^-^, and oxygen saturation (SaO_2_).

### OSA questionnaires

All participants completed the Epworth Sleepiness Scale (ESS) (8), the STOP-Bang questionnaire (9), and the Berlin Questionnaire (10) to categorize them into high-risk and low-risk groups for OSA.

### Echocardiography and cardiac systolic function

Transthoracic echocardiography (TTE) was performed to assess left and right ventricular function. Left ventricular global longitudinal strain (LVGLS) and right ventricular free wall longitudinal strain (RVFWLS) were measured using two-dimensional strain echocardiography (2D-STE).

### Patient grouping

**Figure 1:**
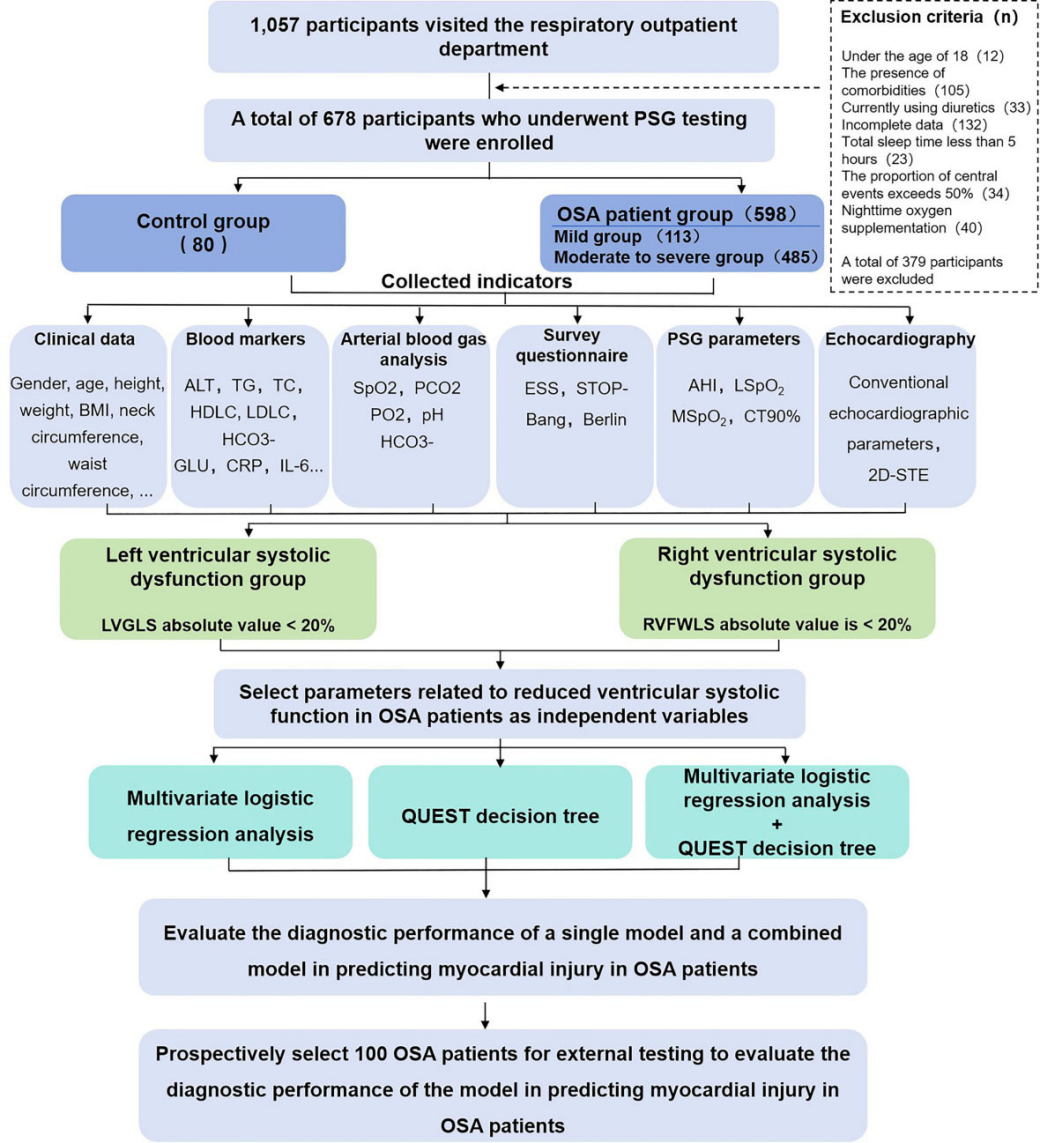
Patient recruitment flow chart for this study.

Patients were grouped into: 1) normal, mild, and moderate-to-severe OSA based on AHI; 2) LVGLS<20% indicating early left ventricular systolic dysfunction (11); and 3) RVFWLS<20% indicating early right ventricular systolic dysfunction (11). The patient recruitment process is shown in Figure 1.

### Ethical statement

We conducted our study in accordance with the Helsinki Declaration of 1975 as revised in 2024. This study was approved by the Institutional Review Board (IRB) of the Third Affiliated Hospital of Anhui Medical University (Hefei First People's Hospital) (approval number 2024-294-01). Because patient data were retrospectively analyzed, the requirement for written informed consent was waived according to the local ethics guidelines. All patient details were de-identified, ensuring anonymity. The reporting of this study conformed to Strengthening the Reporting of Observational Studies in Epidemiology (STROBE) guidelines (12).

### Statistical methods

Data were analyzed using SPSS 19.0 (IBM, USA). Descriptive statistics were used for general information, with normally distributed data reported as means±SD, and non-normally distributed data as median and interquartile range (P_75_-P_25_). Sensitivity and specificity were calculated using a 2×2 contingency table, and the comparison between methods was performed with a paired *t*-test (P<0.05 considered significant).

Logistic regression was performed to identify parameters associated with early myocardial damage (LVGLS<20% or RVFWLS<20%). Significant parameters were treated as independent variables, and LVGLS<20% or RVFWLS<20% were set as dependent variables. Regression coefficients (β) and odds ratios (OR) were calculated to assess the strength and direction of correlations.

The QUEST decision tree model was constructed with a minimum parent node size of 10 and a minimum child node size of 5. Model robustness was assessed using 10-fold cross-validation. The dataset was divided into 10 subsets for training and testing, repeated 10 times.

To assess model performance, we calculated the receiver operating characteristics (ROC) curves and area under the curve (AUC) values for the individual models (logistic regression and QUEST decision tree) and the combined model: for the standalone logistic regression model, the ROC curve was plotted using the logistic regression probability score (P-value) and for the standalone QUEST decision tree model, the ROC curve was derived from the final classification outcome (binary decision: at risk *vs* not at risk).

For the combined model (Logistic + QUEST), we implemented a weighted probability fusion strategy, where the final probability score (*P*_combined_) was computed as a weighted average of the logistic regression and QUEST decision tree outputs by Equation 1:

$$P_{\text{combined}}=\omega_{1}\cdot P_{\text{logistics}}+\omega_{2}\cdot P_{\text{QUEST}}$$ (Eq. 1)

The weights (ω1 and ω2) were optimized using 10-fold cross-validation to maximize the AUC. The final combined probability score was used to generate the ROC curve and compute the AUC for the ensemble model.

Sample size estimation was based on logistic regression and QUEST model requirements. A total of 598 patients were included, meeting the minimum sample size for model stability and validation. The ROC curve analysis indicated the model's predictive performance with an AUC range of 0.78-0.91.

## Results

### Clinical characteristics of participants

A total of 678 participants were included in the study after excluding 379 individuals with coronary artery disease, cancer, non-cooperation, or missing clinical data. Participants were categorized based on the AHI into the control group (80 participants) and the OSA group, which was further divided into mild OSA (113 participants) and moderate-to-severe OSA (485 participants).

In the comparison between the control and OSA groups, the OSA group showed significantly higher BMI, smoking history, hypertension, SBP, DBP, ALT, TG, LDLC, GLU, ESS, STOP-Bang scores, and Berlin Questionnaire scores (P<0.05). No significant differences were found in age, gender, alcohol history, Cr, TC, and HDLC (P>0.05). Between the control and mild OSA groups, the latter had higher BMI, NC, AC, SBP, DBP, ALT, TG, LDL-C, ESS, STOP-Bang scores, and Berlin Questionnaire scores (P<0.05). No significant differences were observed in age, gender, smoking history, alcohol consumption, GLU, TC, and HDLC (P>0.05). In comparison between the mild and moderate-to-severe OSA groups, the latter showed significantly higher BMI, SBP, DBP, hypertension, ALT, TG, GLU and STOP-Bang scores (P<0.05). No significant differences were observed in other parameters (P>0.05). OSA patients exhibited significant metabolic abnormalities in BMI, blood pressure, triglycerides, and fasting glucose, with more severe abnormalities in those with more severe OSA (Table 1).

**Table 1:** General clinical parameters of the study groups.

Parameters	Control group (n=80)	OSA group (n=598)	Mild OSA group (n=113)	Moderate-to-severe OSA group (n=485)	P1	P2
Age (years)	43.04±11.27	43.05±10.34	45.08±10.14	41.94±10.39	0.991	0.489
BMI (kg/m^2^)	23.15±2.68	27.92±4.31	25.94±4.20*	29.01±4.01	<0.001	<0.001
NC (cm)	37.41±3.76	41.93±5.22	40.23±4.05	42.86±4.58	<0.001	<0.001
AC (cm)	115.72±13.68	123.78±18.04	119.25±14.36	126.63±15.49	<0.001	<0.001
Male (n,%)	64 (80.00)	492 (82.27)	90 (79.65)	402 (82.89)	0.732	0.905
History of smoking (n,%)	35 (43.75)	281 (46.99)	52 (46.02)	229 (47.22)	<0.001	0.670
History of alcohol consumption (n,%)	38 (47.50)	269 (44.98)	54 (47.79)	215 (44.33)	0.760	0.198
SBP (mmHg)	125.46±9.72	136.53±13.88	127.32±15.19*	145.70±13.15	<0.001	<0.001
DBP (mmHg)	77.08±7.48	88.29±8.56	83.60±9.41*	93.26±7.44	<0.001	<0.001
Hypertension (%)	22 (27.50)	327 (54.68)	31 (27.43)*	296 (61.03)	<0.001	<0.001
ALT (U/L)	20.00±9.25	26.38±10.67	21.38±12.54*	28.55±10.46	<0.001	<0.001
TG (mmol/L)	1.07±0.31	1.52±1.03	1.16±0.67*	1.65±0.69	<0.001	<0.001
TC (mmol/L)	4.00±0.88	3.95±1.17	4.05±1.11	3.94±1.19	0.802	0.929
HDLC (mmol/L)	1.31±0.42	1.25±0.51	1.26±0.48	1.24±0.59	0.329	0.615
LDLC (mmol/L)	2.78±0.84	3.02±0.91	2.89±0.93	3.09±0.88	<0.05	<0.05
GLU (mmol/L)	4.25±0.44	5.57±0.59	4.28±0.54*	5.93±0.51	<0.001	0.783
Venous HCO_3_^-^ (mmol/L)	24.28±1.46	24.12±2.40	24.09±1.48	24.42±1.43	0.157	0.820
CRP (mg/L)	7.03±3.12	6.88±3.06	6.67±2.86	7.28±2.91	0.687	0.415
IL-6 (pg/mL)	4.28±2.02	4.29±1.84	4.30±1.89	4.31±1.96	0.857	0.971
Ang I (ng/L)	46.56±22.42	48.12±20.06	47.98±19.25	48.52±21.27	0.556	0.647

Data are reported as means and SD. P1: P-value of the comparison between the control group and the OSA patient group; P2: P-value of the comparison between the control group and the mild group; *P<0.05 between mild group and moderate-to-severe group (ANOVA). OSA: obstructive sleep apnea; BMI: body mass index; NC: neck circumference; AC: abdominal circumference; SBP: systolic blood pressure; DBP: diastolic blood pressure; ALT: alanine aminotransferase; TG: triglycerides; TC: total cholesterol; HDLC: high-density lipoprotein cholesterol; LDLC: low-density lipoprotein cholesterol; GLU: glucose; CRP: c-reactive protein; IL-6: interleukin-6.

### PSG results of the enrolled participants

**Figure 2:**
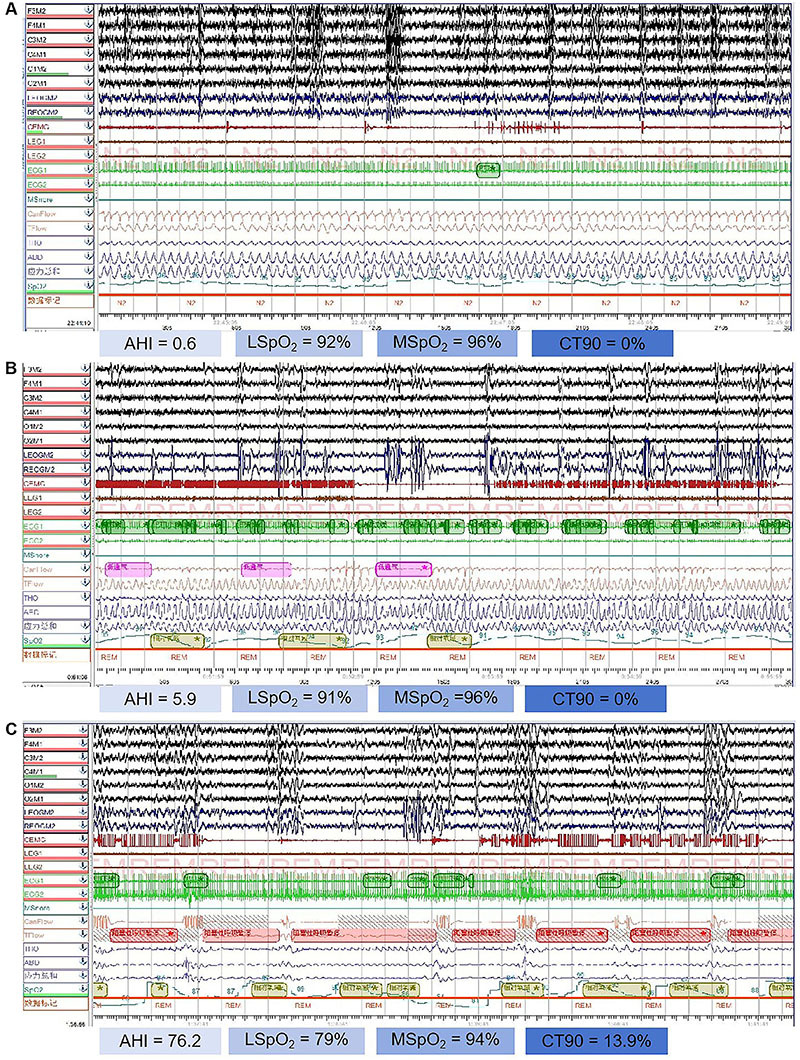
Polysomnography (PSG) test results of participants. **A**, PSG image of a control group participant. **B**, PSG image of a mild obstructive sleep apnea (OSA) patient. **C**, PSG image of a moderate-to-severe OSA patient. PSG: polysomnography; AHI: apnea-hypopnea index; LSpO_2_: lowest oxygen saturation; MSpO_2_: mean oxygen saturation; CT90%: percentage of time with oxygen saturation below 90%.

In this study, significant differences were found by comparing the PSG analysis results of the control group, mild OSA group, and moderate-to-severe OSA group (Figure 2). First, regarding the AHI, there were significant differences between the control group, mild OSA group, and moderate-to-severe OSA group (all P<0.001). Second, regarding lowest (L)SpO_2_, a significant difference was observed between the control group and the moderate-to-severe OSA group (P<0.001), but no statistical difference was found between the control group and the mild OSA group (P=0.094). Third, in terms of mean (M)SpO_2_, significant differences were found between the control group, mild OSA group, and moderate-to-severe OSA group (all P<0.05). Finally, significant differences were also observed in CT90% between the control group, mild OSA group, and moderate-to-severe OSA group (all P<0.05) (Table 2).

**Table 2:** Polysomnography analysis results of the study groups.

Parameters	Control group (n=80)	OSA group (n=598)	Mild OSA group (n=113)	Moderate-to-severe OSA group (n=485)	P1	P2
AHI (mean, SD)	4.9±0.9	42.9±27.0	8.2±4.1	48.2±17.8	<0.001	<0.001
LSpO_2_ (%; P_25_, P_75_)	87.1 (86.0, 92.2)	79.2 (72.0, 85.6)	86.9 (84.0, 88.8)	78.0 (68.8, 84.3)	<0.001	0.094
MSpO_2_ (%; P_25_, P_75_)	97.8 (96.0, 98.0)	94.2 (92.4, 95.8)	95.0 (94.0, 96.0)	94.0 (92.0, 95.0)	<0.001	<0.05
CT90% (%; P_25_, P_75_)	0 (0, 0.05)	7.2 (2.8, 15.2)	0.15 (0, 0.73)	9.6 (4.2, 16.3)	<0.001	<0.05

P1: P-value of the comparison between the control group and the OSA patient group; P2: P-value of the comparison between the control group and the mild group (ANOVA). OSA: obstructive sleep apnea; AHI: apnea hypopnea index; LSpO_2_: lowest pulse oxygen saturation; MSpO_2_: mean pulse oxygen saturation; CT90%: cumulative percentage of the time spent at saturations below 90%.

### Arterial blood gas analysis results

The arterial blood gas analysis results showed that, compared to the control group, the OSA group had significantly higher PaCO_2_ and HCO_3_^-^ levels, along with a lower pH, indicating respiratory dysfunction with carbon dioxide retention and metabolic acidosis. The mild OSA group showed higher HCO_3_^-^ levels than the control group, but there were no significant differences in SaO_2_, PaCO_2_, PaO_2_, and pH, suggesting early compensatory responses in mild OSA patients. Comparing the mild and moderate-to-severe OSA groups, the latter had significantly lower PaO_2_, higher PaCO_2_ and HCO_3_^-^, and a lower pH, reflecting more pronounced respiratory and metabolic abnormalities as OSA severity increased (Table 3).

**Table 3:** Arterial blood gas analysis results of the study groups.

Parameters	Control group (n=80)	OSA group (n=598)	Mild OSA group (n=113)	Moderate-to-severe OSA group (n=485)
SaO_2_ (%)	97.5±2.1	97.3±2.0	97.5±2.2	96.8±2.1
PaCO_2_ (mmHg)	37.62±5.86	39.25±6.07	38.64±6.01*	40.52±6.11*
PaO_2_ (mmHg)	86.41±5.62	86.02±5.99	86.67±6.02*	84.13±5.88*
pH	7.40±0.04	7.36±0.04	7.39±0.04*	7.34±0.03*
HCO_3_^-^ (mmol/L)	23.04±1.42	26.63±1.64	25.88±1.47*	26.98±1.80*

Data are reported as means and SD. *P<0.05 between groups (ANOVA). OSA: obstructive sleep apnea; SaO_2_: arterial oxygen saturation; PaCO_2_: arterial carbon dioxide partial pressure; PaO_2_: arterial partial pressure of oxygen; pH: potential of hydrogen; HCO_3_^-^: bicarbonate.

### Echocardiographic findings across study groups

**Figure 3:**
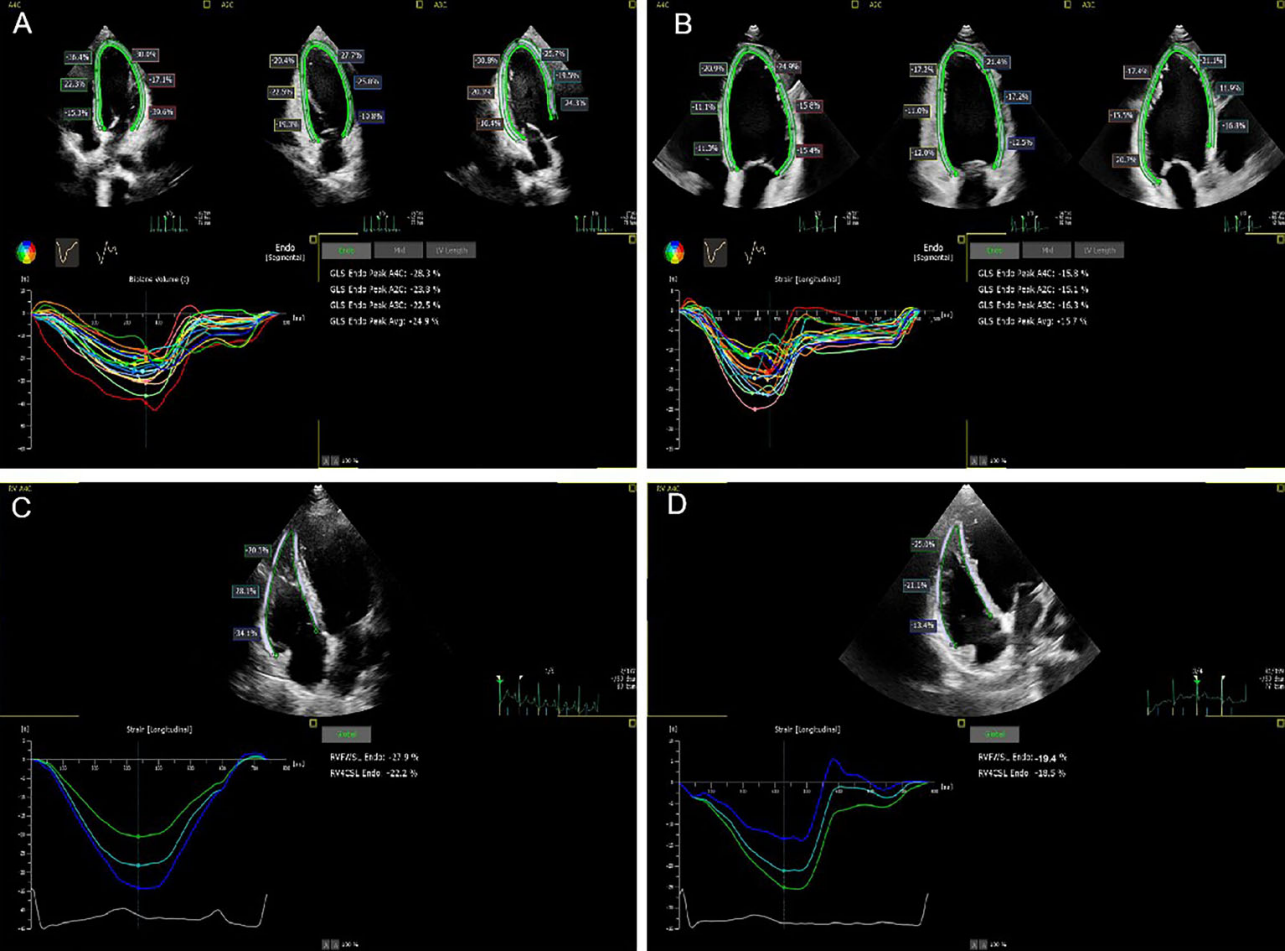
Two-dimensional strain echocardiography measurement of LVGLS and RVFWLS in obstructive sleep apnea patients. **A**, Upper image showing multiple cross-sectional views of the left ventricle, with green lines marking the endocardial contours. LVGLS=-24.9% and RVFWLS=-27.9%, indicating normal left and right ventricular systolic function. **B**, Ultrasound images showing cross-sectional views of the left ventricle, with LVGLS=-15.7% and RVFWLS=-19.4%, indicating impaired left and right ventricular systolic function. **C**, Cross-sectional view of the right ventricular free wall showing LVGLS=-24.9% and RVFWLS=-27.9%, indicating normal left and right ventricular systolic function. **D**, Cross-sectional view of the right ventricular free wall showing LVGLS=-15.7% and RVFWLS=-19.4%, indicating reduced function in both ventricles. LVGLS: left ventricular global longitudinal strain; RVFWLS: right ventricular free wall longitudinal strain.

This study compared the left ventricular (LV) parameters among the control, mild OSA, and moderate-to-severe OSA groups. While left ventricular end-diastolic dimension (LVEDD), left ventricular end-systolic dimension (LVESD), left ventricular posterior wall thickness (LVPWT), and left ventricular end-diastolic volume (LVEDV) were within normal ranges for all groups, the interventricular septum diameter (IVSD) exceeded normal limits in the moderate-to-severe OSA group, indicating interventricular septal thickening. Additionally, LVGLS was reduced across all OSA patients, reflecting impaired left ventricular systolic function (Figure 3A and B).

Control *vs* OSA group: the OSA group showed significantly larger LVEDD, LVESD, IVSD, LVPWT, LVEDV, and left ventricular end-systolic volume (LVESV), indicating left ventricular dilation and increased volume load (P1<0.05). Left ventricular ejection fraction (LVEF) and LVGLS were significantly decreased, suggesting impaired systolic function (P1<0.05). Control *vs* mild OSA group: the mild OSA group had a significantly higher (LVEF) (P2<0.05) but a significantly lower LVGLS (P2<0.05), indicating early changes in left ventricular systolic function. Mild *vs* moderate-to-severe OSA group: the moderate-to-severe OSA group showed significantly larger LVEDD, LVESD, IVSD, LVPWT, LVEDV, and LVESV (P<0.001), with significantly reduced LVEF and LVGLS (P<0.001), reflecting progressive deterioration of LV function (Table 4).

**Table 4:** Comparison of left ventricular echocardiographic data among the study groups.

Parameters	Control group (n=80)	OSA group (n=598)	Mild OSA group (n=113)	Moderate-to-severe OSA group (n=485)	P1	P2
LVEDD (mm)	46.53±2.31	47.89±2.42	45.08±2.14*	48.81±3.55	<0.001	0.082
LVESD (mm)	27.28±2.79	28.92±2.19	25.24±3.20*	29.51±3.01	<0.001	0.056
IVSD (mm)	8.60±1.13	9.62±2.24	8.61±0.94*	10.60±1.16	<0.001	0.032
LVPWT (mm)	8.36±1.80	8.66±1.28	8.37±1.17*	8.92±1.28	<0.05	0.221
LVEDV (mL)	88.55±20.49	95.69±15.46	90.27±13.28*	98.29±11.91	<0.001	0.061
LVESV (mL)	34.51±2.95	36.35±3.72	34.00±3.71*	38.74±4.73	<0.001	0.057
LVEF (%)	68.74±5.57	65.53±4.72	68.29±4.80*	62.55±4.65	<0.001	<0.05
LVGLS (%)	23.94±2.44	18.94±2.33	19.60±2.66^*^	17.64±2.51	<0.001	<0.05

Data are reported as means and SD. P1: P-value of the comparison between the control group and the OSA patient group; P2: P-value of the comparison between the control group and the mild group; *P<0.05 mild group compared with moderate-to-severe group (ANOVA). OSA: obstructive sleep apnea; OSA: LVDEE: left ventricular end-diastolic diameter; LVESD: left ventricular end-systolic diameter; IVSD: interventricular septum thickness in end-diastole; LVPWT: left ventricular posterior wall thickness in end-diastole; LVEDV: left ventricular end-diastolic volume; LVESV: left ventricular end-systolic volume; LVEF: left ventricular ejection fraction; LVGLS: left ventricular global longitudinal strain.

For right ventricular (RV) parameters, although right ventricular basal diameter (RVBD), right ventricular fractional area change (RVFAC), tricuspid annular plane systolic excursion (TAPSE), and tissue doppler imaging-derived systolic peak velocity (TDI-S') in the OSA group were within normal limits, RVBD was significantly larger, and RVFAC, TAPSE, TDI-S', tricuspid regurgitation velocity (TRV), pulmonary artery systolic pressure (PASP), and RVFWLS were significantly impaired compared to the control group (P1<0.001), indicating RV dilation and systolic dysfunction (Figure 3C and D).

Control *vs* mild OSA group: the mild OSA group exhibited significantly increased RVBD (P2<0.001), and significantly decreased RVFAC, TAPSE, TDI-S', TRV, PASP, and RVFWLS (P2<0.001), indicating right ventricular dysfunction even in mild OSA. Mild *vs* moderate-to-severe OSA group: in the moderate-to-severe OSA group, all RV parameters were significantly worsened (P<0.001), confirming progressive right ventricular systolic impairment with increasing OSA severity (Table 5).

**Table 5:** Comparison of right ventricular systolic function of the study groups.

Parameters	Control group (n=80)	OSA group (n=598)	Mild OSA group (n=113)	Moderate-to-severe OSA group (n=485)	P1	P2
RVBD (mm)	35.44±3.24	36.87±3.23	35.59±3.65*	38.17±4.13	<0.001	<0.001
RVFAC (%)	51.53±6.09	43.76±5.79	44.52±5.46*	38.66±4.72	<0.001	<0.001
TAPSE (mm)	22.37±3.67	20.00±2.30	22.05±1.87*	19.01±2.70	<0.001	0.526
TDI-S**'** (cm/s)	13.44±2.31	12.28±1.72	13.23±1.34*	11.05±1.71	<0.001	<0.001
TRV (m/s)	1.17±0.88	1.19±0.74	1.17±0.86*	1.21±0.87	<0.001	0.217
PASP (mmHg)	17.85±6.64	18.49±5.95	17.86±5.71*	18.94±6.16	<0.001	0.266
RVFWLS (%)	33.72±5.32	18.20±6.87	20.90±5.23*	19.06±5.87	<0.001	<0.001

Data are reported as means and SD. P1: P-value of the comparison between the control group and the OSA patient group; P2: P-value of the comparison between the control group and the mild group; *P<0.05 mild group compared with moderate-to-severe group (ANOVA). OSA: obstructive sleep apnea; VBD: right ventricular base diameter; RVFAC: right ventricle fractional area change; TAPSE: tricuspid annular plane systolic excursion; TDI-S**'**: tissue Doppler imaging; TRV: tricuspid regurgitation velocity; PASP: pulmonary artery systolic pressure; RVFWLS: right ventricle free wall longitudinal strain.

### Factors influencing early left and right ventricular systolic dysfunction in OSA patients

#### Left ventricular systolic dysfunction

Among OSA patients, 99 had normal LVGLS and 499 had reduced LVGLS. In the mild OSA group, 24 had normal LVGLS and 89 had reduced LVGLS; in the moderate-to-severe OSA group, 75 had normal LVGLS and 410 had reduced LVGLS. Comparison of parameters revealed that patients with reduced LVGLS had significantly higher BMI (P<0.001), larger NC (P<0.001), and greater AC (P<0.05) compared to those with normal LVGLS. No significant differences were found in terms of SBP, DBP, hypertension prevalence, liver function, lipid metabolism markers, GLU, and questionnaire scores. PSG analysis showed significant differences in AHI (P<0.05), MSpO_2_, and CT90% (both P<0.001), with arterial blood HCO_3_^-^ levels significantly higher in the reduced LVGLS group (P<0.001), indicating a compensatory response to respiratory acidosis. No significant differences were observed in conventional echocardiographic parameters, but LVEF was significantly lower in the reduced LVGLS group (P<0.05), though both groups remained within the normal range (Table 6).

**Table 6:** Comparison of various data between the normal LVGLS group and the reduced LVGLS group in obstructive sleep apnea patients.

Parameters	Normal LVGLS (n=99)	Reduced LVGLS (n=499)	P
Age (years)	43.04±9.27	43.06±10.34	0.98
BMI (kg/m^2^)	25.15±5.68	29.95±4.31	<0.001
NC (cm)	41.33±4.55	42.88±3.98	<0.001
AC (cm)	121.22±22.16	125.13±27.49	<0.05
Male (n, %)	82 (82.82)	410 (82.16)	0.98
History of smoking (n, %)	46 (44.44)	235 (45.09)	0.99
History of alcohol consumption (n, %)	44 (48.00)	225 (63.01)	0.61
SBP (mmHg)	135.46±19.72	136.89±22.18	0.55
DBP (mmHg)	87.08±7.22	89.29±9.56	0.03
Hypertension (n, %)	55 (55.56)	272 (54.51)	0.94
ALT (u/L)	26.38±10.11	27.01±12.77	0.64
TG (mmol/L)	1.45±0.67	1.59±0.69	0.06
TC (mmol/L)	4.01±1.04	3.85±1.22	0.22
HDLC (mmol/L)	1.26±0.71	1.25±0.81	0.91
LDLC (mmol/L)	3.02±0.77	3.02±0.89	0.92
GLU (mmol/L)	5.53±0.47	5.60±0.58	0.68
Venous bicarbonate (mmol/L)	24.11±2.21	24.13±2.43	0.94
CRP (mg/L)	6.86±4.06	6.89±3.54	0.94
IL-6 (Pg/mL)	4.29±1.94	4.30±1.24	0.95
Ang I (ng/L)	48.02±22.16	48.32±11.06	0.84
AHI	41.9±21.0	42.2±22.0	<0.05
LSpO_2_ (%; P_25_, P_75_)	85.9 (84.0, 88.8)	84.2 (72.0, 85.6)	0.07
MSpO_2_ (%; P_25_, P_75_)	94.8 (93.4, 96.0)	93.1 (91.9, 95.8)	<0.001
CT90% (%; P_25_, P_75_)	1.7 (0, 4.73)	7.6 (5.6, 16.3)	<0.001
Arterial SaO_2_ (%)	97.5±4.7	97.4±8.9	0.91
Arterial PaCO_2_ (%)	39.95±7.07	40.05±9.44	0.92
Arterial PaO_2_ (%)	87.07±9.92	86.97±8.02	0.91
Arterial pH	7.37±0.06	7.36±0.05	0.30
Arterial bicarbonate (mmol/L)	25.52±2.47	27.01±1.90	<0.001
LVEDD (mm)	47.44±4.52	47.92±7.61	0.82
LVESD (mm)	28.56±7.19	28.97±5.11	0.06
IVSD (mm)	9.59±2.33	9.63±1.89	0.58
LVPWT (mm)	8.61±1.48	8.67±2.31	0.22
LVEDV (mL)	95.21±11.77	95.89±19.46	0.06
LVESV (mL)	36.05±5.12	36.45±3.98	0.78
LVEF (%)	66.07±6.01	65.63±5.55	<0.05
ESS Positive (n, %)	80 (80.81)	411 (82.46)	0.78
STOP-Bang Positive (n, %)	93 (93.94)	464 (92.99)	0.89
Berlin Positive (n, %)	76 (76.77)	402 (80.56)	0.33

Data are reported as means and SD or median and interquartile range (*t-*test or Mann-Whitney *U* test). BMI: body mass index; NC: neck circumference; AC: abdominal circumference; SBP: systolic blood pressure; DBP: diastolic blood pressure; ALT: alanine aminotransferase; TG: triglycerides; TC: total cholesterol; HDLC: high-density lipoprotein cholesterol; LDLC: low-density lipoprotein cholesterol; GLU: glucose; CRP: c-reactive protein; IL-6: interleukin-6; AHI: apnea hypopnea index; LSpO_2_: lowest pulse oxygen saturation; MSpO_2_: mean pulse oxygen saturation; CT90%: cumulative percentage of the time spent at saturations below 90%; LVDEE: left ventricular end-diastolic diameter; LVESD: left ventricular end-systolic diameter; IVSD: interventricular septum thickness in end-diastole; LVPWT: left ventricular posterior wall thickness in end-diastole; LVEDV: left ventricular end-diastolic volume; LVESV: left ventricular end-systolic volume; LVEF: left ventricular ejection fraction.

#### Right ventricular systolic dysfunction

A total of 86 OSA patients had normal RVFWLS and 512 had reduced RVFWLS. Among the mild OSA group, 22 had normal RVFWLS and 91 had reduced RVFWLS; in the moderate-to-severe OSA group, 64 had normal RVFWLS and 421 had reduced RVFWLS. The BMI of the reduced RVFWLS group was significantly higher than the normal group (P<0.001), while NC and AC were also significantly larger in the reduced RVFWLS group (P<0.05). There were no significant differences in smoking history, alcohol consumption, or hypertension prevalence. Blood gas analysis showed significantly higher arterial HCO_3_^-^ levels in the reduced RVFWLS group (P<0.001). PSG parameters showed differences in AHI (P<0.05) and significant variations in MSpO_2_ and CT90% (both P<0.001). However, conventional right heart echocardiographic parameters (RVBD, RVFAC, TAPSE, TDI-S', TRV, and PASP) did not differ significantly between the two groups (P>0.05) (Table 7).

**Table 7:** Comparison of various data between the normal RVFWLS group and the reduced RVFWLS group in obstructive sleep apnea patients.

Parameters	Normal RVFWLS (n=86)	Reduced RVFWLS (n=512)	P
Age (years)	42.94±9.73	43.18±10.11	0.83
BMI (kg/m^2^)	26.25±7.68	28.95±9.44	<0.001
NC (cm)	41.77±3.22	42.09±11.29	<0.05
AC (cm)	122.98±28.16	124.03±23.08	<0.05
Male (n, %)	72 (83.72)	420 (82.03)	0.610
History of smoking (n,%)	40 (46.51)	241 (47.07)	0.910
History of alcohol consumption (n,%)	39 (45.34)	230 (44.92)	0.942
SBP (mmHg)	136.06±21.02	135.79±24.22	0.931
DBP (mmHg)	88.08±7.31	87.97±10.21	0.618
Hypertension (%)	46 (53.38)	281 (54.88)	0.810
ALT (u/L)	25.98±9.81	26.71±14.77	0.422
TG (mmol/L)	1.51±0.96	1.52±0.77	0.734
TC (mmol/L)	3.95±1.31	3.95±1.22	0.769
HDLC (mmol/L)	1.26±0.51	1.25±0.77	0.914
LDLC (mmol/L)	3.01±0.56	3.03±0.67	0.870
GLU (mmol/L)	5.55±0.49	5.59±0.59	0.76
Venous bicarbonate (mmol/L)	24.11±3.41	24.12±4.55	0.995
CRP (mg/L)	6.87±4.46	6.89±5.54	0.966
IL-6 (Pg/mL)	4.28±2.33	4.29±2.24	0.863
Ang I (ng/L)	47.93±25.16	48.22±19.86	0.973
AHI	41.9±25.0	42.1±22.0	<0.05
LSpO_2_ (%; P_25_, P_75_)	80.1 (76.8, 82.9)	79.2 (71.9, 83.9)	0.677
MSpO_2_ (%; P_25_, P_75_)	96.8 (93.4, 97.8)	94.6 (89.9, 95.4)	<0.001
CT90% (%; P_25_, P_75_)	2.05 (0, 4.73)	9.2 (5.6, 17.9)	<0.001
Arterial SaO_2_ (%)	97.6±5.6	97.3±6.7	0.912
Arterial PaCO_2_ (%)	39.85±9.07	40.25±10.17	0.930
Arterial PaO_2_ (%)	87.27±10.44	85.97±9.88	0.987
Arterial pH	7.37±0.06	7.37±0.07	0.711
Arterial bicarbonate (mmol/L)	24.72±2.56	26.76±2.60	<0.001
RVBD (mm)	36.57±6.22	36.89±4.01	0.823
RVFAC (%)	43.96±4.55	43.01±3.19	0.343
TAPSE (mm)	20.10±3.30	19.91±2.89	0.743
TDI-S' (cm/s)	12.27±2.11	12.28±3.34	0.983
TRV (m/s)	1.18±0.44	1.19±0.78	0.926
PASP (mmHg)	18.79±6.25	18.06±5.78	0.478
ESS Positive (n, %)	68 (79.07)	423 (82.62)	0.242
STOP-Bang Positive (n,%)	81 (94.19)	476 (92.97)	0.837
Berlin Positive (n, %)	68 (79.07)	410 (80.08)	0.808

Data are reported as means and SD unless otherwise informed (*t-*test or Mann-Whitney *U* test). BMI: body mass index; NC: neck circumference; AC: abdominal circumference; SBP: systolic blood pressure; DBP: diastolic blood pressure; ALT: alanine aminotransferase; TG: triglycerides; TC: total cholesterol; HDLC: high-density lipoprotein cholesterol; LDLC: low-density lipoprotein cholesterol; GLU: glucose; CRP: c-reactive protein; IL-6: interleukin-6; AHI: apnea hypopnea index; LSpO_2_: lowest pulse oxygen saturation; MSpO_2_: mean pulse oxygen saturation; CT90%: cumulative percentage of the time spent at saturations below 90%; RVBD: right ventricular base diameter; RVFAC: right ventricle fractional area change; TAPSE: tricuspid annular plane systolic excursion; TDI-S**'**: tissue Doppler imaging; TRV: tricuspid regurgitation velocity; PASP: pulmonary artery systolic pressure.

### Logistic regression prediction of early left and right ventricular systolic dysfunction risk in OSA patients

**Figure 4:**
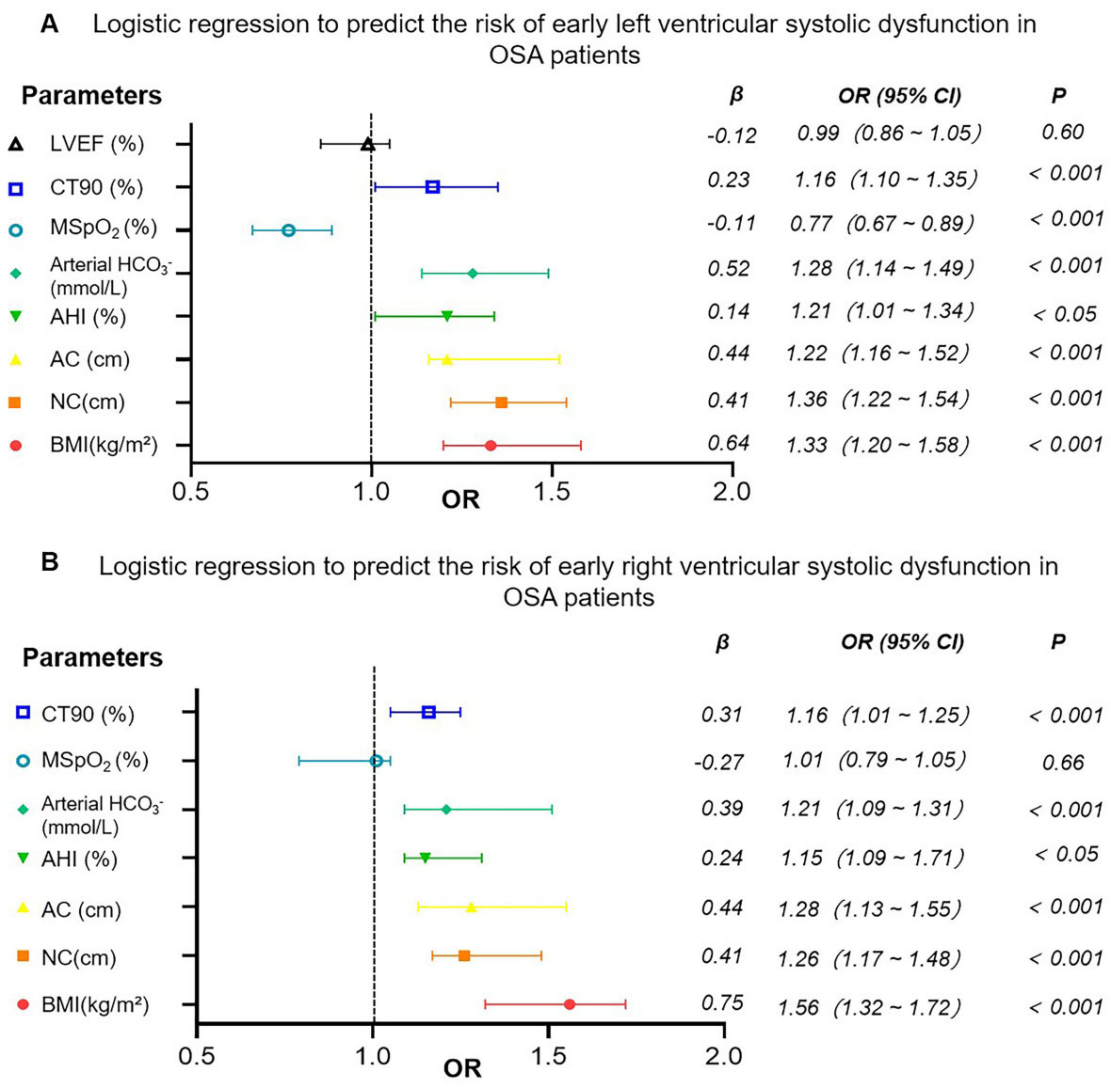
Logistic regression analysis of early left and right ventricular systolic dysfunction in obstructive sleep apnea (OSA) patients. The vertical axis represents variables included in the logistic regression analysis and the horizontal axis shows the odds ratio (OR) and 95% confidence interval (CI). **A**, Early left ventricular systolic dysfunction: percentage of sleep time with oxygen saturation <90% (CT90%), mean oxygen saturation (MSpO_2_), arterial blood HCO_3_^-^, apnea-hypopnea index (AHI), abdominal circumference (AC), neck circumference (NC), and body mass index (BMI) were significant independent predictors (P<0.05). LVEF did not show a significant correlation (P=0.60). **B**, Early right ventricular systolic dysfunction: CT90, AHI, AC, NC, and BMI were significant independent predictors (P<0.05). MSpO_2_ did not show a significant correlation (P=0.66). LVEF: left ventricular ejection fraction.

Multivariate logistic regression was conducted to predict the risk of early left and right ventricular systolic dysfunction in OSA patients. For early left ventricular systolic dysfunction (defined as LVGLS<20%), significant parameters included BMI, neck circumference, waist circumference, AHI, arterial blood HCO_3_^-^, MspO_2_, and CT90%, all of which were significantly correlated with reduced LVGLS. LVEF did not show a significant correlation with early left ventricular systolic dysfunction (P=0.60) (Figure 4A).

For early right ventricular systolic dysfunction (defined as RVFWLS<20%), significant parameters included BMI, neck circumference, waist circumference, AHI, arterial blood HCO_3_^-^, and CT90%, all of which were significantly correlated with reduced RVFWLS. MSpO_2_ did not show a significant correlation with the occurrence of early right ventricular systolic dysfunction (P=0.66) (Figure 4B).

### Prediction of early left and right ventricular systolic dysfunction in OSA patients using the QUEST decision tree

The QUEST decision tree was used to predict the risk of early left and right ventricular systolic dysfunction in OSA patients based on significant parameters.

**Figure 5:**
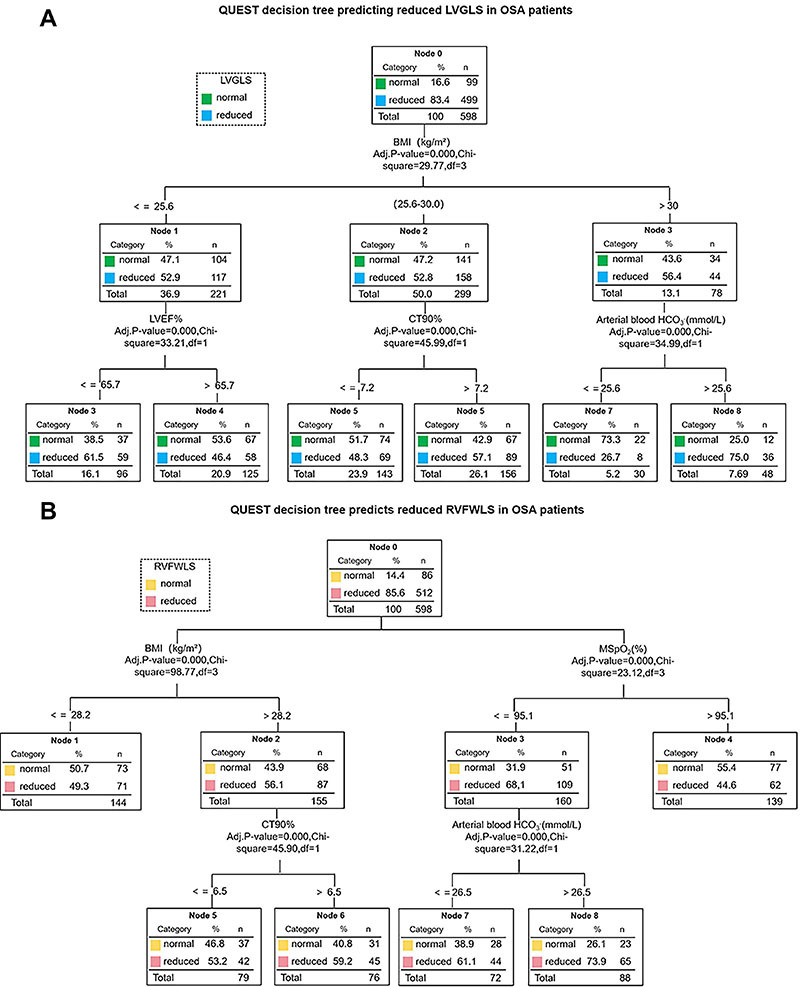
QUEST decision tree-based prediction models for left ventricular global longitudinal strain (LVGLS) and right ventricular free wall longitudinal strain (RVFWLS) reduction in obstructive sleep apnea (OSA) patients. **A**, The primary splitting variable for LVGLS reduction was body mass index (BMI), followed by mean oxygen saturation (MSpO_2_), percentage of sleep time with oxygen saturation <90% (CT90%), and arterial blood HCO_3_^-^. Each node represents the proportion and sample size, demonstrating how each variable stratifies the risk for LVGLS reduction. **B**, The primary splitting variables for RVFWLS reduction were BMI and MspO_2_, followed by CT90% and arterial blood HCO_3_^-^. Each node represents the proportion and sample size, showing how each variable influences the risk for RVFWLS reduction.

For early left ventricular systolic dysfunction (LVGLS<20%), the key nodes were BMI, arterial blood HCO_3_^-^, LVEF, and CT90%. BMI was the first splitting variable, with patients having higher BMI (>0) showing a 93.4% risk of LVGLS reduction (Node 3). In patients with BMI between 25.6 and 30.0, CT90% further divided the samples, with those having CT90%>7.2 showing 57.1% LVGLS reduction (Node 5). For patients with BMI ≤25.6, LVEF was the main splitting factor, with those having LVEF≤65.7% showing 61.5% LVGLS reduction (Node 3). Additionally, for patients with BMI>30, arterial blood HCO_3_^-^>25.6 was associated with 75.0% LVGLS reduction (Node 8) (Figure 5A).

For early right ventricular systolic dysfunction (RVFWLS<20%), the key nodes were BMI, arterial blood HCO_3_^-^, MspO_2_, and CT90%. BMI was the first splitting variable, with patients having higher BMI (>28.2) showing 56.1% RVFWLS reduction (Node 2). In patients with BMI>28.2, MSpO_2_ was a significant factor, with MSpO_2_≤95.1 associated with 68.1% RVFWLS reduction (Node 3). For those with BMI≤28.2, CT90 divided the samples, with those having CT90≤6.5 showing 53.2% RVFWLS reduction (Node 5). Additionally, in patients with MSpO_2_≤95.1, arterial blood HCO_3_^-^>26.5 was linked to 73.9% RVFWLS reduction (Node 8) (Figure 5B).

### Prediction of early left and right ventricular systolic dysfunction in OSA patients based on logistic regression combined with the QUEST decision tree

Combining the results from logistic regression analysis and the QUEST decision tree, key parameters associated with early left and right ventricular systolic dysfunction were identified and used to construct predictive models for LVGLS and RVFWLS reduction in OSA patients.

**Figure 6:**
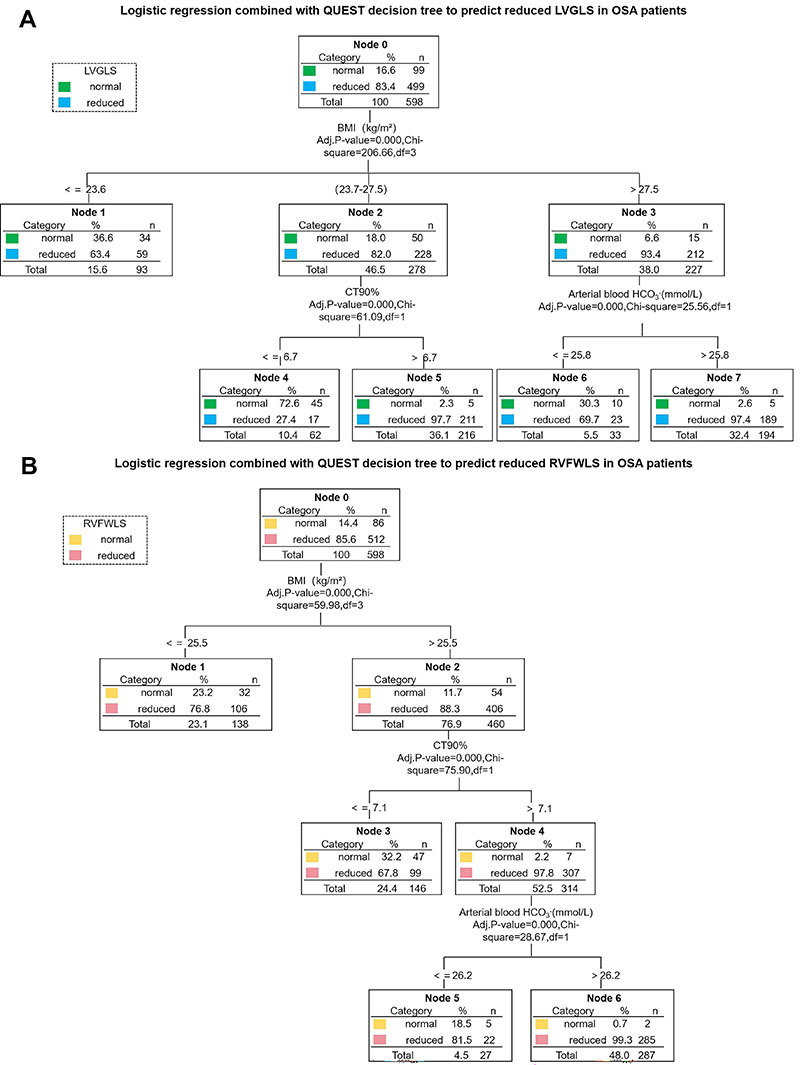
Logistic and QUEST combined models for predicting left ventricular global longitudinal strain (LVGLS) and right ventricular free wall longitudinal strain (RVFWLS) reduction in obstructive sleep apnea (OSA) patients. **A**, Decision tree for predicting LVGLS reduction based on key variables (BMI, arterial blood HCO_3_^-^, CT90%) identified by logistic regression and QUEST decision tree. The model stratifies risk with BMI as the primary variable, followed by arterial blood HCO_3_^-^ and CT90%. Each node shows proportions for normal and reduced LVGLS. **B**, Decision tree for predicting RVFWLS reduction based on QUEST decision tree. BMI is the primary splitting variable, followed by CT90% and arterial blood HCO_3_^-^, highlighting their roles in risk stratification. Each node shows proportions for normal and reduced RVFWLS, illustrating the impact of each variable on risk.

For early left ventricular systolic dysfunction (LVGLS<20%), BMI, arterial blood HCO_3_^-^, and CT90% were selected as key parameters. BMI was the primary splitting variable (P<0.001, χ^2^=76.12), dividing the samples into two groups: BMI≤26.2 (Node 1) and BMI>26.2 (Node 2). The LVGLS reduction rate was 41.9% in the BMI≤26.2 group and 67.3% in the BMI>26.2 group. In patients with BMI>26.2, arterial blood HCO_3_^-^ further refined the samples (P<0.001, χ^2^=34.89), with 58.4% of those with HCO_3_^-^≤25.8 showing LVGLS reduction (Node 3) compared to 81.6% of those with HCO_3_^-^>25.8 (Node 4). In patients with BMI≤26.2, CT90% was the next splitting variable (P<0.001, χ^2^=29.03), with 56.2% of those with CT90%>7.1 showing LVGLS reduction (Node 5), while only 33.7% of those with CT90%≤7.1 had LVGLS reduction (Node 6) (Figure 6A).

For early right ventricular systolic dysfunction (RVFWLS<20%), BMI, arterial blood HCO_3_^-^, and CT90% were also identified as key parameters. BMI was the first splitting variable (P<0.001, χ^2^=59.98), dividing the samples into BMI≤25.5 (Node 1) and BMI>25.5 (Node 2). The RVFWLS reduction rate was 76.8% in the BMI≤25.5 group and 88.3% in the BMI>25.5 group. In patients with BMI>25.5, CT90% further split the samples (P<0.001, χ^2^=75.90), with 67.8% of those with CT90%≤7.1 showing RVFWLS reduction (Node 3) compared to 97.8% of those with CT90%>7.1 (Node 4). In patients with CT90%>7.1, arterial blood HCO_3_^-^ further split the samples (P<0.001, χ^2^=28.67), with 81.5% of those with HCO_3_^-^≤26.2 showing RVFWLS reduction (Node 5), compared to 99.3% of those with HCO_3_^-^>26.2 (Node 6) (Figure 6B).

### Evaluation of predictive performance of single and combined models for early myocardial contractile dysfunction in OSA patients

**Figure 7:**
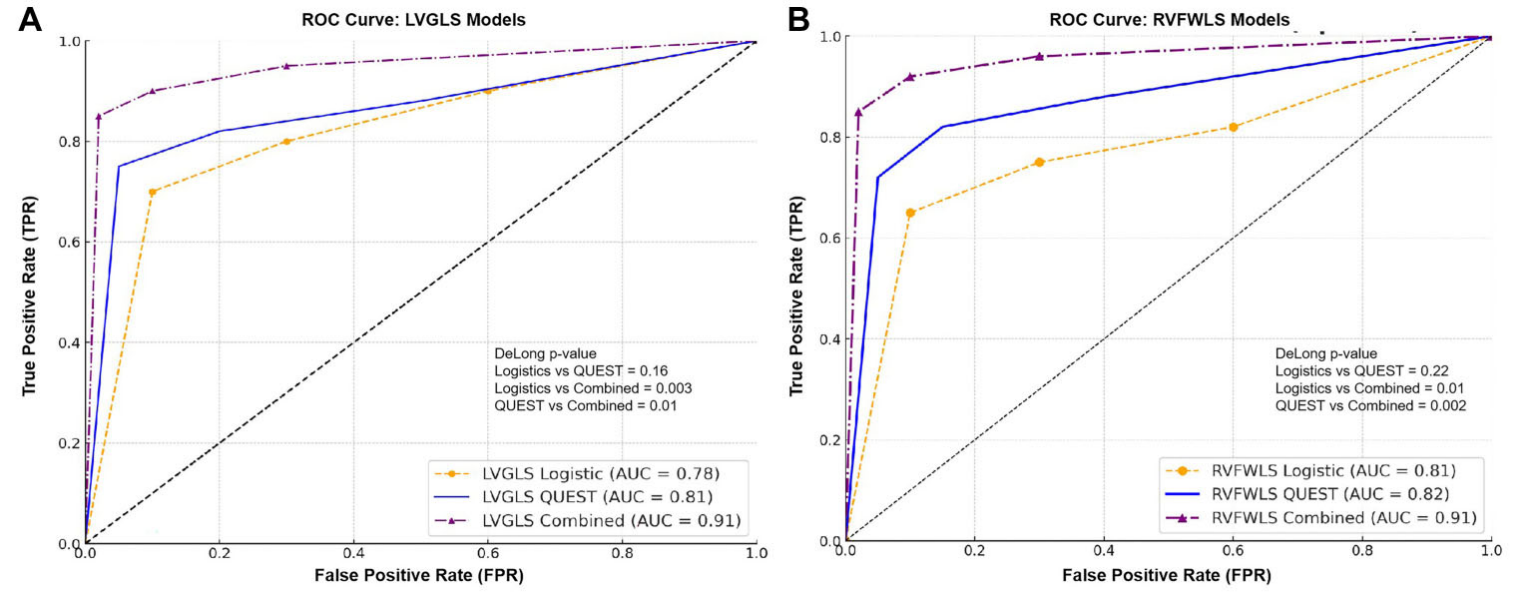
Evaluation of diagnostic performance of models in predicting early myocardial impairment in obstructive sleep apnea (OSA) patients. Panels show the ROC curves and area under the curve (AUC) values for the logistic regression model, QUEST decision tree model, and their combined model in predicting left ventricular global longitudinal strain (LVGLS) (**A**) and right ventricular free wall longitudinal strain (RVFWLS) (**B**) reduction.

To assess the predictive performance of single and combined models for early myocardial contractile dysfunction in OSA patients, we evaluated the logistic regression model, the QUEST decision tree model, and their combined model using ROC curve analysis. For predicting LVGLS reduction, the logistic regression model yielded an AUC of 0.78, while the QUEST decision tree model showed an improved AUC of 0.81, demonstrating its superior predictive ability. The combined model outperformed both, achieving the highest AUC of 0.91. Similarly, for predicting RVFWLS reduction, the logistic regression model had an AUC of 0.81, the QUEST decision tree model had an AUC of 0.82, and the combined model again demonstrated optimal performance with an AUC of 0.91, confirming its best predictive capability (Figure 7).

### External validation of the combined logistic regression and QUEST decision tree model in predicting myocardial injury in OSA patients

To further validate the applicability and effectiveness of the combined logistic regression and QUEST decision tree model for predicting early myocardial injury in OSA patients, an external testing study was conducted.

#### Sample source

The external testing sample included 100 OSA patients prospectively recruited from the respiratory sleep clinic of the Third Affiliated Hospital of Anhui Medical University, consisting of 36 mild and 64 moderate-to-severe cases to ensure representation across various severity levels.

#### Key parameters

Data on key variables related to the model, including BMI, CT90%, arterial blood HCO_3_^-^, as well as LVGLS and RVFWLS from echocardiography, were collected.

#### Statistical analysis

A random sample of 100 cases from the original dataset, matched to the external testing sample, was used for comparison. Ten parameters - BMI, neck circumference, waist circumference, AHI, MspO_2_, CT90%, arterial blood HCO_3_^-^, LVEF, LVGLS, and RVFWLS - were analyzed using Bland-Altman plots to assess the consistency between the external testing and internal datasets. ROC curve analysis evaluated the diagnostic performance of each model, and the DeLong test compared the AUC differences between the models.

#### Results

Compared to the mild OSA group, the moderate OSA group showed significantly higher BMI, waist circumference, AHI, CT90%, and HCO_3_^-^ (P<0.001), along with significantly reduced LVGLS and RVFWLS (P<0.05) (Table 8). In the comparison between the normal and reduced LVGLS groups, BMI, waist circumference, AHI, CT90%, and HCO_3_^-^ were significantly higher in the LVGLS reduction group (P<0.001) (Table 9). A similar pattern was observed between the normal and reduced RVFWLS groups, with significantly higher values of BMI, waist circumference, AHI, CT90%, and HCO_3_^-^ in the RVFWLS reduction group (P<0.001) (Table 10).

**Table 8:** Severity grouping of obstructive sleep apnea patients in the external dataset.

Parameters	Mild OSA group (n=36)	Moderate-to-severe OSA group (n=64)	P
Age (years)	47.47±8.86	50.48±11.52	0.85
Male (n,%)	32 (88.9)	57 (89.1)	0.98
BMI (kg/m^2^)	23.12±4.54	26.91±4.03	<0.01
NC (cm)	39.87±4.15	42.66±4.47	<0.001
AC (cm)	118.63±15.20	124.93±14.49	<0.001
AHI	9.9 (11.5, 26.8)	25.5 (13.3, 29.9)	<0.05
MSpO_2_ (%; P_25_, P_75_)	89.6 (92.7, 96.3)	89.5 (89.5, 95.5)	<0.001
CT90% (%; P_25_, P_75_)	3.6 (0.4, 6.0)	8.3 (6.2, 16.6)	<0.001
Arterial bicarbonate (mmol/L)	24.82±1.57	26.01±1.78	<0.001
LVEF (%)	67.88±4.70	63.15±4.55	<0.001
LVGLS (%)	21.4±1.8	19.2±3.3	<0.05
RVFWLS (%)	22.0±3.5	18.9±3.9	<0.01

Data are reported as means and SD (*t*-test or chi-squared test). BMI: body mass index; NC: neck circumference; AC: abdominal circumference; AHI: apnea hypopnea index; MSpO_2_: mean pulse oxygen saturation; CT90%: cumulative percentage of the time spent at saturations below 90%; LVEF: left ventricular ejection fraction; LVGLS: left ventricular global longitudinal strain; RVFWLS: right ventricle free wall longitudinal strain.

**Table 9:** Comparison of various data between the normal and reduced LVGLS groups in obstructive sleep apnea patients from the external dataset.

Parameters	Normal LVGLS (n= 59)	Reduced LVGLS (n=41)	P
Age (years)	43.04±9.27	43.06±10.34	0.98
Male (n, %)	52 (88.1)	37 (90.2)	0.74
BMI (kg/m^2^)	25.15±5.68	29.95±4.31	<0.001
NC (cm)	40.52±4.55	42.13±4.21	<0.001
AC (cm)	120.23±16.31	123.06±14.31	<0.001
AHI	41.9±21.0	42.2±22.0	<0.05
MSpO_2_ (%; P_25_, P_75_)	94.8 (92.4, 96.0)	93.1 (91.9, 95.8)	<0.001
CT90% (%; P_25_, P_75_)	1.7 (0.0, 4.73)	7.6 (5.6, 16.3)	<0.001
Arterial bicarbonate (mmol/L)	25.04±1.76	25.93±1.68	<0.001
LVEF (%)	66.11±5.03	64.89±4.43	<0.001

Data are reported as means and SD (*t*-test, chi-squared test, or Mann-Whitney *U* test). BMI: body mass index; NC: neck circumference; AC: abdominal circumference; AHI: apnea hypopnea index; MSpO_2_: mean pulse oxygen saturation; CT90%: cumulative percentage of the time spent at saturations below 90%; LVEF: left ventricular ejection fraction.

**Table 10:** Comparison of various parameters between the normal and reduced RVFWLS groups in the external dataset of obstructive sleep apnea patients.

Parameters	Normal RVFWLS (n=56)	Reduced RVFWLS (n=44)	P
Age (years)	42.12±8.93	43.50±10.87	0.85
Male (n,%)	50 (89.3)	39 (88.6)	0.92
BMI (kg/m^2^)	24.67±5.12	30.10±4.25	<0.001
NC (cm)	40.43±4.48	42.17±4.23	<0.001
AC (cm)	120.79±16.35	122.86±14.13	<0.001
AHI	40.5±20.7	43.1±22.5	<0.05
MSpO_2_ (%; P_25_, P_75_)	95.2 (93.8, 97.0)	92.9 (91.4, 94.5)	<0.001
CT90% (%; P_25_, P_75_)	2.3 (0.0, 5.0)	8.1 (6.2, 17.0)	<0.001
Arterial bicarbonate (mmol/L)	24.93±1.66	25.98±1.72	<0.001

Data are reported as means and SD, n and %, or median and interquartile range (t-test, chi-squared test, or Mann-Whitney *U* test). BMI: body mass index; NC: neck circumference; AC: abdominal circumference; AHI: apnea hypopnea index; MSpO_2_: mean pulse oxygen saturation; CT90%: cumulative percentage of the time spent at saturations below 90%.

**Figure 8:**
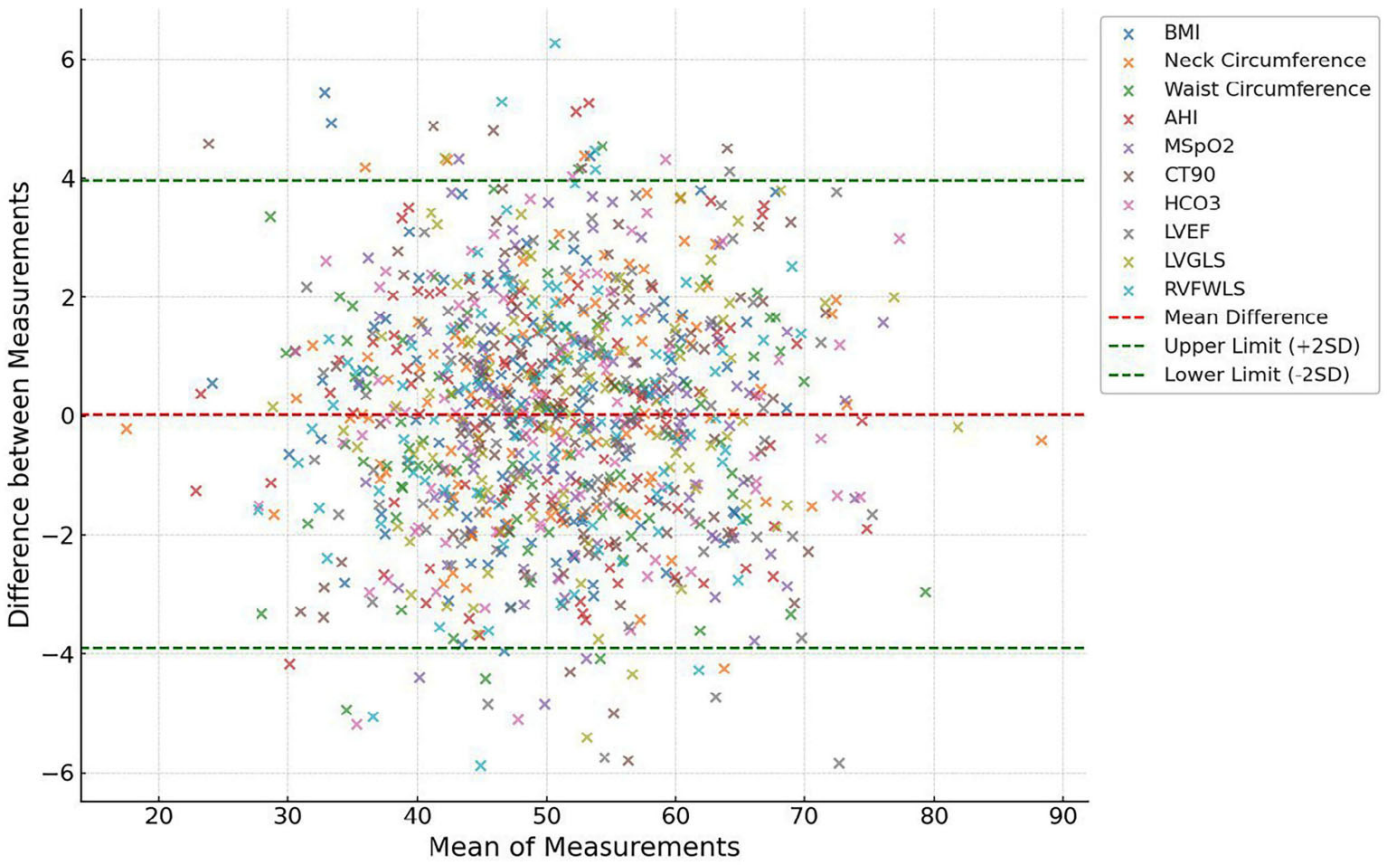
Bland-Altman analysis plots for each parameter in the external test set. The red dashed line represents the mean difference (bias) and the green dashed lines indicate the upper and lower limits (±2SD). The plots demonstrate that differences between external parameters and the internal test dataset fall within the predetermined range of agreement, indicating high consistency. BMI: body mass index; AHI: apnea hypopnea index; MSpO_2_: mean pulse oxygen saturation; CT90%: cumulative percentage of the time spent at saturations below 90%; LVEF: left ventricular ejection fraction; LVGLS: left ventricular global longitudinal strain; RVFWLS: right ventricular free wall longitudinal strain.

The Bland-Altman plot (Figure 8) was used to demonstrate the consistency between the external testing set and the internal dataset. The plot centered around the ±2SD range, indicating good overall consistency of parameters between the two datasets. The mean difference (red dashed line) for each parameter was close to zero, reflecting minimal systemic bias, and most points fell between the -2SD and +2SD lines, suggesting high measurement consistency. However, certain parameters, such as AHI and CT90%, showed some points near or beyond the ±2SD range, indicating possible larger measurement discrepancies or extreme values in specific cases.

**Figure 9:**
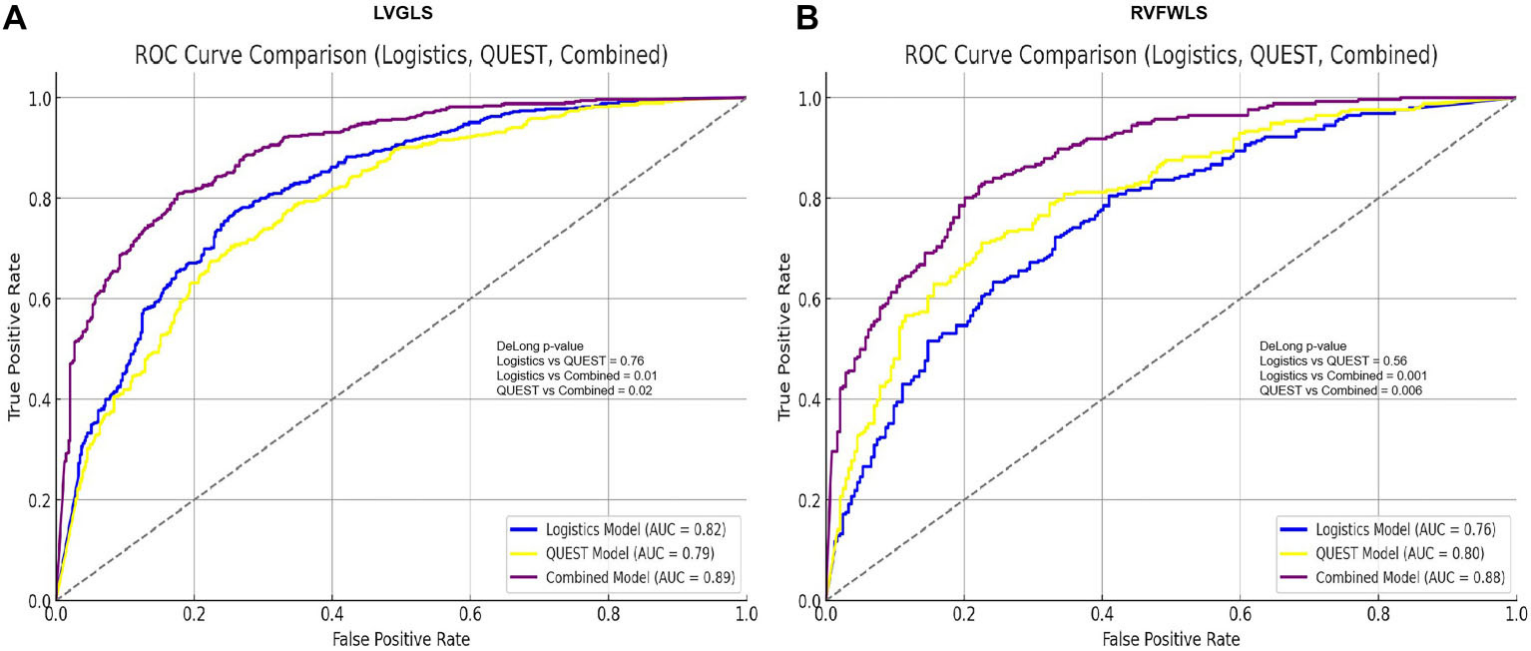
Diagnostic performance of logistic regression, QUEST decision tree, and combined models in predicting left ventricular global longitudinal strain (LVGLS) and right ventricular free wall longitudinal strain (RVFWLS) reduction in obstructive sleep apnea (OSA) patients. **A**, ROC curves for predicting LVGLS reduction. **B**, ROC curves for predicting RVFWLS reduction.

Diagnostic performance (Figure 9) revealed that for the prediction of LVGLS reduction, the AUC of the logistic regression model was 0.82, while the AUC for the QUEST decision tree model was 0.79. The combined model showed a significantly higher AUC of 0.89. DeLong test results indicated no significant difference between the logistic regression and QUEST models (P=0.76), but both models showed statistically significant differences when compared to the combined model (P=0.01 and P=0.02), demonstrating the superior predictive performance of the combined model.

For the prediction of RVFWLS reduction, the AUC for the logistic regression model was 0.76, the QUEST decision tree model had an AUC of 0.80, and the combined model achieved an AUC of 0.88. The DeLong test showed no significant difference between the logistic regression and QUEST models (P=0.56), but both models showed significantly lower AUC values compared to the combined model (P=0.001 and P=0.006), further confirming the optimal performance of the combined model in predicting myocardial injury in OSA patients.

## Discussion

This study provides a comprehensive evaluation of myocardial function in patients with OSA and explores the factors influencing early myocardial contractile dysfunction. By developing a combined predictive model using logistic regression and the QUEST decision tree, the study offers valuable insights into the mechanisms underlying myocardial injury in OSA and highlights the potential for early intervention and precision treatment.

Artificial intelligence (AI) techniques have already been applied to OSA, primarily focusing on predicting obstructive sleep apnea syndrome using data sources other than the gold standard (PSG), such as anthropometric indices, trachea/respiratory sounds, imaging data, ECG, EEG, oximetry, and screening questionnaires. AI has also been employed to predict treatment outcomes, evaluate treatment efficacy, and personalize treatment (13-17). Our study represents the first application of AI in the early detection of myocardial dysfunction potentially induced by OSA.

Our findings demonstrated significant impairment in both LVGLS and RVFWLS in OSA patients, with this dysfunction worsening as the severity of the disease increases. Moderate-to-severe OSA patients exhibited significantly lower LVGLS and RVFWLS compared to mild OSA patients, though some decline was already evident in the mild group. This suggests that myocardial dysfunction may begin in the early stages of OSA, and that more pronounced dysfunction occurs as the disease progresses (18-20). These results support previous studies that have identified early myocardial dysfunction in OSA and emphasize the importance of early identification and timely intervention to prevent further damage to myocardial function.

From a mechanistic perspective, myocardial dysfunction in OSA is likely driven by a combination of factors (21). The recurrent and intermittent nocturnal hypoxia characteristic of OSA leads to systemic oxidative stress and inflammatory responses, which can directly damage myocardial cells (22,23). Additionally, OSA patients often exhibit metabolic abnormalities, such as increased BMI and lipid metabolism disorders (24,25), which further exacerbate structural and functional myocardial damage. This study identified BMI, CT90%, and arterial blood HCO_3_^-^ as independent predictors of myocardial dysfunction in OSA patients. In particular, increased BMI and prolonged CT90% reflected the core role of obesity and hypoxia in driving myocardial injury, underscoring the importance of managing these risk factors in OSA patients.

To better understand the relationships between these complex factors, we employed a combined approach using multivariable logistic regression and QUEST decision tree analysis. Logistic regression helped quantify the independent contributions of key variables, such as BMI, CT90%, and arterial blood HCO_3_^-^, to myocardial dysfunction. For instance, a 1 kg/m^2^ increase in BMI was associated with a significantly higher risk of LVGLS reduction, while elevated levels of arterial blood HCO_3_^-^ suggested that the patient's compensatory metabolic mechanisms may be overwhelmed. In contrast, the QUEST decision tree revealed nonlinear relationships and interaction effects between variables, providing a more nuanced understanding of how factors such as BMI and CT90% interact to increase myocardial risk. This dual approach, combining linear and nonlinear models, not only enhanced the predictive accuracy of our model but also improved its interpretability and clinical applicability (26).

In terms of diagnostic performance, the combined model outperformed both the logistic regression and QUEST decision tree models alone. The AUC for predicting LVGLS and RVFWLS reductions was 0.91 for the combined model, significantly higher than the AUCs of 0.78 and 0.81 for the logistic regression and QUEST models, respectively. This suggests that integrating the strengths of both models provides a more comprehensive assessment of the multiple factors influencing myocardial dysfunction in OSA, improving the accuracy and reliability of risk stratification. By more accurately identifying high-risk patients, the combined model offers valuable guidance for clinicians in tailoring treatment strategies (27).

External validation further supported the robustness and generalizability of the combined model. The AUCs for the external dataset remained high, demonstrating that the model's predictive performance was stable across different populations and clinical settings. This suggests that the combined model has strong potential for real-world applications, extending beyond the internal dataset used in this study.

This study also underscored the clinical significance of monitoring key indicators such as BMI, CT90%, and arterial blood HCO_3_^-^ in OSA patients. These variables not only exhibit high reproducibility and measurability but also reflect the overall health status and disease progression. For example, patients with elevated BMI may benefit from proactive weight management, including dietary interventions and exercise, to reduce cardiovascular risk and improve sleep quality (28). For patients with elevated CT90%, intensified CPAP therapy could help alleviate hypoxia and reduce myocardial strain (29). Furthermore, dynamic monitoring of arterial blood HCO_3_^-^ levels may provide valuable insights into a patient's metabolic status, guiding therapeutic decisions and optimizing treatment outcomes (30).

However, several limitations must be acknowledged. The study sample primarily consisted of symptomatic OSA patients from a sleep clinic, with a higher proportion of moderate-to-severe cases. This may introduce sample bias, limiting the generalizability of our findings to patients with mild OSA. Additionally, the study was cross-sectional in design, and no long-term follow-up was conducted to track changes in myocardial function over time, limiting our ability to draw causal inferences. Furthermore, the underlying mechanisms of some variables, such as the increase in arterial blood HCO_3_^-^, require further investigation to clarify whether it was a direct result of metabolic compensation or influenced by other factors. Finally, while we did observe patients with biventricular dysfunction in our study, this investigation specifically focused on early myocardial dysfunction in OSA. Given the exceptionally low prevalence of biventricular impairment in the mild OSA subgroup (insufficient sample size) and its predominant association with moderate-to-severe cases, no further mechanistic investigations or in-depth analyses were conducted on this subset.

Future research should address these limitations by expanding the sample size and validating the model's generalizability through multicenter studies. Longitudinal studies would also help explore temporal changes in myocardial function and their relationship with therapeutic interventions in OSA patients. Moreover, further basic research is needed to elucidate the mechanistic roles of key variables, providing a more solid theoretical foundation for personalized treatment. Finally, incorporating advanced machine learning and AI techniques into predictive models could further enhance their clinical utility and accuracy.

In conclusion, this study developed an integrated predictive model that effectively assessed early myocardial dysfunction in OSA patients by combining logistic regression and the QUEST decision tree. The study highlights the critical roles of BMI, CT90%, and arterial blood HCO_3_^-^ as predictive factors, offering important insights for early risk stratification and personalized treatment. The superior diagnostic performance of the combined model suggests it has strong potential for clinical application. With further optimization and validation, this model could become an essential tool for improving the long-term health outcomes of OSA patients.
